# Cardiac Lesions and Initial Laboratory Data in Kawasaki Disease: a Nationwide Survey in Japan

**DOI:** 10.2188/jea.JE20140128

**Published:** 2015-03-05

**Authors:** Masanari Kuwabara, Mayumi Yashiro, Kazuhiko Kotani, Satoshi Tsuboi, Ryusuke Ae, Yosikazu Nakamura, Hiroshi Yanagawa, Tomisaku Kawasaki

**Affiliations:** 1 Department of Public Health, Jichi Medical University, Shimotsuke, Tochigi, Japan; 1 自治医科大学 公衆衛生学教室; 2 Department of Cardiology, Toranomon Hospital, Tokyo, Japan; 2 虎の門病院 循環器センター内科; 3 Japan Kawasaki Disease Research Center, Tokyo, Japan; 3 日本川崎病研究センター

**Keywords:** mucocutaneous lymph node syndrome, coronary disease, coronary aneurysm, valvular lesion, epidemiology

## Abstract

**Background:**

Cardiac lesions, such as coronary dilatation, aneurysms, narrowing, myocardial infarction, and valvular lesions, sometimes occur in Kawasaki disease, but most studies have only evaluated cardiac lesions in the later phase of the disease. This study was undertaken to clarify the related factors between cardiac lesions and laboratory data in the initial phase of Kawasaki disease.

**Methods:**

We conducted a cross-sectional study using data for 26 691 patients from the 22nd nationwide survey of Kawasaki disease in Japan, the observation period of which was from January 2011 through December 2012. We excluded patients with recurrent Kawasaki disease and who were more than seven days from the start of symptoms at admission. We analyzed 23 155 cases (13 353 boys; mean age: 923 ± 734 days) with available laboratory data for white blood cell count, platelet count, serum albumin, and C-reactive protein (CRP).

**Results:**

Cardiac lesions were detected in 984 cases (656 boys and 328 girls); lesions were classified as coronary dilatation (764 cases), coronary aneurysm (40), giant coronary aneurysm (6), coronary narrowing (3), and valvular lesions (204). The significant related factors of initial coronary dilatation were male sex (odds ratio [OR] 1.73), older age (OR per 100 days increase 1.03), higher platelet count (OR per 10 000 cells/µL increase 1.006), lower albumin (OR per 1 g/dL increase 0.66), and higher CRP (OR per 1 mg/dL increase 1.02). The factors related to coronary aneurysm were higher platelet count (OR 1.01) and lower albumin (OR 0.34). No factors were significantly related to giant coronary aneurysm. The related factors of valvular lesions were age (OR 0.98), and higher CRP (OR 1.05).

**Conclusions:**

Clinicians should consider male sex, older age, higher platelet count, lower albumin levels, and higher CRP levels when assessing risk of cardiac lesions in the initial phase of Kawasaki disease.

## Abstract (ja)

**背景：:**

川崎病では冠動脈拡張、動脈瘤、心筋梗塞、弁膜病変などの心臓病変が時々認められる。これまでの川崎病における心臓病変の評価は、急性期から慢性期にかけて行われたものがほとんどである。本研究は各種血液検査データを含む川崎病初診時の状態と、心臓病変との関係性について明らかにした。

**方法：:**

本邦で2011年1月から2012年12月までに行われた、第22回川崎病全国調査の26691例の患者データを用いて横断研究を行った。本研究では、入院時に川崎病の症状出現から8日以内で、各種血液検査（白血球数、血小板数、血清アルブミン、C反応性タンパク（CRP））を行っている23155例（男児13353例、平均日齢：923±734日）を対象とした。

**結果：:**

心臓病変は984例（男児656例）に認められ、内訳は冠動脈拡張（764例）、冠動脈瘤（40例）、巨大冠動脈瘤（6例）、冠動脈狭窄（3例）、弁膜病変（204例）であった。冠動脈拡張の有意な関連因子は、加齢（100日増加ごとにオッズ比（OR）：1.034）、男性（OR：1.731）、高血小板数（10000 /μL増加ごとにOR：1.006）、低アルブミン（1 g/ dL増加ごとにOR：0.659）、高CRP（1 mg/ dL増加ごとにOR：1.023）であった。冠状動脈瘤の有意な関連因子は、高血小板（10000 /μL増加ごとにOR：1.010）、低アルブミン（1 g/ dL増加ごとにOR：0.337）であった。巨大冠動脈瘤の有意な関連要因は認めなかった。弁膜病変の有意な関連因子は、日齢（100日増加ごとにOR：0.977）、高CRP（1 mg/ dL増加ごとにOR：1.045）であった。

**結論：:**

川崎病の初診時において、心臓病変を見逃さないために、男性、年齢、高血小板数、低アルブミン、高CRPに注意を払うことが必要である。

## INTRODUCTION

Kawasaki disease was first reported in Japanese with clinical observation of 50 cases by Dr. Kawasaki in 1967.^1^ The disease was reported in English as mucocutaneous lymph node syndrome (MLNS) in 1974.^2^ After myocardial infarction in Kawasaki disease was reported in 1974,^3^ many reports showed that cardiac lesions, such as coronary dilatation, coronary aneurysms, coronary narrowing, myocardial infarction, and valvular lesions, often occur in patients with Kawasaki disease.^4^^–^^6^ Cardiac lesions, which can be detected by echocardiography, are serious complications of Kawasaki disease.^7^^–^^9^ The proportion of coronary lesions in untreated patients was 25%,^10^ but treatment with intravenous immunoglobulin improves clinical outcomes and reduces the proportion of coronary lesions to less than 5%.^11^ Several previous studies examined the risk factors for cardiac lesion in patients with Kawasaki disease,^12^^,^^13^ especially for giant coronary aneurysms.^14^^,^^15^ The prevalence of cardiac lesions and clinical parameters associated with cardiac lesions have been determined in the later phase of Kawasaki disease. However, data on the prevalence of cardiac lesions and clinical parameters associated with cardiac lesions in the early phase is inadequate.

The number of patients with Kawasaki disease has been increasing worldwide.^16^^–^^19^ It is important for patients with Kawasaki disease not to delay treatment with intravenous immunoglobulin after diagnosis. We conducted this study to determine the relationship between cardiac lesions in patients with Kawasaki disease and laboratory data at admission using a nationwide epidemiologic survey of Kawasaki disease in Japan. The purpose of the present study is to determine relevant information regarding the ‘initial phase’ of Kawasaki disease and confirm the prevalence of classified cardiac lesions as well as clinical parameters (such as age, gender, and laboratory data) associated with cardiac lesions in these patients. It is crucial to know the prevalence of classified cardiac lesions. Early management of cardiac lesions in patients with Kawasaki disease is possible using data from the initial check-up of clinical parameters. We already know that patients with Kawasaki disease are more likely to be male and have higher white blood cell (WBC) counts, higher C-reactive protein (CRP) levels, higher platelet counts, and lower albumin levels.^20^ Kawasaki disease is a systemic vasculitis, and these factors are strongly associated with vasculitis. We hypothesize that these risk factors were also associated with coronary lesions. This survey has been conducted nearly every 2 years since 1970, and several features of Kawasaki disease have been revealed.^21^^–^^25^ The most recent survey, the 22nd, was undertaken in 2013.

## METHODS

We conducted a cross-sectional study using data from the 22nd nationwide survey of Kawasaki disease in Japan, the target patients of which were surveyed from January 2011 through December 2012. The medical facilities asked to participate in this survey were hospitals specializing in pediatrics and hospitals with a total of 100 or more beds and a pediatric department.

Patient information requested on the questionnaire was name (only initials), address (municipality), sex, date of birth, and date of illness at first hospital visit, presence or absence of acute cardiac lesions, and laboratory data such as WBC count, platelet count, serum albumin, and CRP at admission.

Cardiac lesions in this study were classified at admission. All patients were diagnosed with cardiac lesions based on findings from two-dimensional echocardiography. We classified cardiac lesions as coronary dilatation, coronary aneurysm, giant coronary aneurysm, coronary narrowing, myocardial infarction, and valvular lesions. We used criteria for cardiac lesions in Kawasaki disease as defined by the Japanese Ministry of Health.^26^ Coronary dilatation was defined as maximum absolute internal lumen diameter >3 mm in children aged younger than 5 years or >4 mm in children aged 5 years or older. Coronary aneurysm was defied as a segmental internal diameter of any segment ≥1.5 times greater than that of an adjacent segment. Coronary narrowing was defined as the presence of a clearly irregular coronary artery. Giant coronary aneurysm was defined as an internal lumen diameter >8 mm. We used patient data to clarify factors related to cardiac lesions in Kawasaki disease. Exclusion criteria were: recurrent Kawasaki disease, and more than eight days lapsed since the start of symptoms at the time of admission. After checking for possible inconsistencies on the questionnaires, the forms were sent back to the respondents to correct any errors. The Ethics Board of Jichi Medical University approved this survey in advance (November 2012).

### Statistical analysis

Statistical analyses were performed using SPSS Statistics software (IBM SPSS Statistics version 19 for Windows; IBM, Armonk, NY, USA). The statistically significant level was set at α = 0.05. Related factors of cardiac lesions in Kawasaki disease were clarified by univariate and multivariate analysis. Multivariate analysis was conducted by logistic regression analyses with adjustment for age, sex, and laboratory values, including WBC count, platelet count, serum albumin level, and CRP level.

## RESULTS

Data were available for 26 691 patients (mean age: 954 ± 761 days, 57.9% male). Data regarding cardiac lesions was unavailable for 252 patients, and laboratory data was unavailable for 1555 patients. We excluded 885 cases of recurrent Kawasaki disease and 844 cases for which symptoms began more than eight days prior to admission. Therefore, we analyzed 23 155 cases (86.8%; 57.7% male, mean age: 923 ± 734 days). The mean day of illness at first hospital visit was 3.8 ± 1.4 days (range: 0–7 days). Mean WBC count was 14 634 × 10^3^ ± 5763 × 10^3^ cells/µL (range: 1000 × 10^3^–194 200 × 10^3^ cells/µL), mean platelet count was 33.5 × 10^4^ ± 11.8 × 10^4^ cells/µL (2.1 × 10^4^–426.0 × 10^4^ cells/µL), mean serum albumin level was 3.81 ± 0.44 g/dL (1.0–8.8 g/dL), and mean CRP level was 7.57 ± 4.97 g/dL (0.01–58.00 g/dL).

Cardiac lesions at first hospital visit were detected in 984 cases (4.25%), including 656 boys (4.91%) and 328 girls (3.35%). We classified cardiac lesions as coronary dilatation (764 cases, 3.30%), coronary aneurysm (40 cases, 0.17%), giant coronary aneurysm (6 cases, 0.026%), coronary narrowing (3 cases, 0.013%), myocardial infarction (0 cases, 0%), and valvular lesions (204 cases, 0.88%) (Figure). The factors related to initial coronary dilatation in Kawasaki disease was male sex (odds ratio [OR]: 1.73; 95% confidence interval [CI]: 1.48–2.03), older age (OR per 100 days increase: 1.03; 95% CI: 1.02–1.04), higher platelet count (OR per 10 000 cells/µL increase: 1.006, 95% CI: 1.001–1.01), lower albumin level (OR per 1 g/dL increase: 0.66; 95% CI: 0.56–0.78), and higher CRP level (OR per 1 mg/dL increase: 1.02; 95% CI: 1.01–1.04) (Table 1). The factors related to coronary aneurysm were higher platelet count (OR per 10 000 cells/µL increase: 1.01; 95% CI: 1.001–1.02), lower albumin level (OR per 1 g/dL increase: 0.34; 95% CI: 0.17–0.65) (Table 2). No factors were significantly related to giant coronary aneurysm (Table 3). The related factors of coronary narrowing and myocardial infarction could not be calculated due to a paucity of cases. The factors related to valvular lesions were older age (OR per 100 days increase: 0.98; 95% CI: 0.96–0.999), and higher CRP level (OR per 1 mg/dL increase: 1.05; 95% CI: 1.02–1.07) (Table 4).

**Figure.**
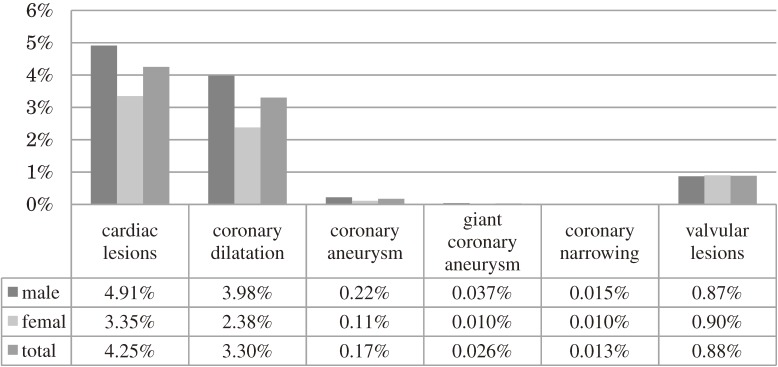
The prevalence of classified cardiac lesions in Kawasaki disease.

**Table 1.** Factors related to coronary dilatation in Kawasaki disease

Coronary dilatation	Univariate			Multivariate^a^		
	
	Odds ratio	95% CI	*P*	Odds ratio	95% CI	*P*
Sex (male)	1.701	1.455–1.988	<0.001	1.731	1.480–2.025	<0.001
Age (per 100 days increased)	1.034	1.026–1.043	<0.001	1.033	1.024–1.042	<0.001
White blood cell count (per 1000/µl increased)	1.008	0.998–1.019	0.119	1.001	0.989–1.014	0.823
Platelet count (per 10 000/µl increased)	1.002	0.997–1.008	0.387	1.006	1.001–1.011	0.011
Albumin level (per 1 g/dl increased)	0.588	0.500–0.691	<0.001	0.659	0.557–0.779	<0.001
C-reactive protein level (per 1 mg/dl increased)	1.042	1.028–1.055	<0.001	1.023	1.009–1.038	0.002

CI, confidence interval.

^a^Multivariate analysis was conducted by logistic regression analyses with age, sex, and laboratory data such as such as white blood cell count, platelet count, serum albumin and C-reactive protein.

**Table 2.** Factors related to coronary aneurysm in Kawasaki disease

Coronary aneurysm	Univariate			Multivariate^a^		
	
	Odds ratio	95% CI	*P*	Odds ratio	95% CI	*P*
Sex (male)	1.937	0.967–3.880	0.062	1.929	0.962–3.869	0.064
Age (per 100 days increased)	1.004	0.963–1.046	0.853	0.996	0.952–1.041	0.858
White blood cell (per 1000/µl increased)	1.020	0.994–1.047	0.131	1.013	0.976–1.050	0.504
Platelet (per 10 000/µl increased)	1.012	1.004–1.020	0.004	1.010	1.001–1.020	0.035
Albumin (per 1 g/dl increased)	0.275	0.148–0.511	<0.001	0.337	0.174–0.652	0.001
C-reactive protein (per 1 mg/dl increased)	1.069	1.019–1.122	0.007	1.038	0.985–1.095	0.165

CI, confidence interval.

^a^Multivariate analysis was conducted by logistic regression analyses with age, sex, and laboratory data such as such as white blood cell count, platelet count, serum albumin and C-reactive protein.

**Table 3.** Factors related to giant coronary aneurysm in Kawasaki disease

Giant coronary aneurysm	Univariate			Multivariate^a^		
	
	Odds ratio	95% CI	*P*	Odds ratio	95% CI	*P*
Sex (male)	3.671	0.429–31.430	0.235	3.700	0.429–31.885	0.234
Age (per 100 days increased)	1.060	0.984–1.142	0.127	1.048	0.966–1.138	0.257
White blood cell (per 1000/µl increased)	1.012	0.917–1.117	0.815	0.968	0.823–1.139	0.698
Platelet (per 10 000/µl increased)	1.008	0.973–1.043	0.663	1.009	0.982–1.037	0.515
Albumin (per 1 g/dl increased)	0.131	0.043–0.753	0.019	0.366	0.067–1.994	0.245
C-reactive protein (per 1 mg/dl increased)	1.141	1.053–1.236	0.001	1.101	0.994–1.219	0.064

CI, confidence interval.

^a^Multivariate analysis was conducted by logistic regression analyses with age, sex, and laboratory data such as such as white blood cell count, platelet count, serum albumin and C-reactive protein.

**Table 4.** Factors related to valvular disease in Kawasaki disease

Valvular lesions	Univariate			Multivariate^a^		
	
	Odds ratio	95% CI	*P*	Odds ratio	95% CI	*P*
Sex (male)	0.967	0.732–1.278	0.815	0.970	0.734–1.281	0.828
Age (per 100 days increased)	0.989	0.969–1.009	0.278	0.977	0.955–0.999	0.036
White blood cell (per 1000/µl increased)	1.004	0.982–1.026	0.721	0.999	0.973–1.026	0.950
Platelet (per 10 000/µl increased)	0.995	0.982–1.009	0.489	0.993	0.978–1.007	0.325
Albumin (per 1 g/dl increased)	0.705	0.517–0.963	0.028	0.795	0.577–1.096	0.162
C-reactive protein (per 1 mg/dl increased)	1.043	1.018–1.068	0.001	1.045	1.017–1.073	0.001

CI, confidence interval.

^a^Multivariate analysis was conducted by logistic regression analyses with age, sex, and laboratory data such as such as white blood cell count, platelet count, serum albumin and C-reactive protein.

## DISCUSSION

This study is one of the largest-scale assessments of the relationship between cardiac lesions and laboratory data in patients with Kawasaki disease. We know that Kawasaki disease patients with cardiac lesions are more likely to be older, male, and to have higher WBC count, CRP levels, and platelet count, as well as lower albumin levels.^20^ This study showed that those same factors were associated with coronary dilatation in patients with Kawasaki disease. These risk factors of cardiac lesions were very similar to the risk factors of Kawasaki disease observed in past studies. Only WBC count was not detected as a risk factor in this study. These results support the hypothesis that the severity of Kawasaki disease is associated with progression of cardiac lesions. Higher levels of inflammation are associated with coronary damage. A previous report showed that older age was a risk factor for the development of cardiac lesions in Kawasaki disease.^12^ Another report showed that long duration of fever before treatment and low serum albumin level at hospital admission were factors that predisposed patients with Kawasaki disease to cardiac lesions.^27^ Since Kawasaki disease is an acute systemic vasculitis, it is reasonable to treat patients with Kawasaki disease using intravenous immunoglobulin or intravenous methylprednisolone in order to prevent cardiac lesions.^28^^,^^29^

This study had some limitations. First, this study was cross-sectional and retrospective, so it was difficult to detect the intimate relationship between the cardiac lesions and laboratory data. We could not explain whether cardiac lesions induce the differences in laboratory data or whether these laboratory data (inflammation due to vasculitis) induce cardiac lesions. The laboratory data might be confounding factors of vasculitis and coronary lesions of Kawasaki disease patients. Second, it was difficult to determine the previous cardiac condition of these patients before the onset of Kawasaki disease. We were sure to include only patients without past history of Kawasaki disease, but there was a possibility that some patients had cardiac lesions before the onset of Kawasaki disease. Third, this study was conducted only in Japan, and it may be difficult to apply these findings to patients in other countries. Finally, there were relatively few cases of coronary aneurysm (40 cases) and giant coronary aneurysm (6 cases), which made it difficult to show a significant difference between incidence of these two conditions. However, the related factors of coronary aneurysm and giant coronary aneurysm were very similar to those of coronary dilatation.

Cardiac lesions are serious complications of Kawasaki disease. Male sex, older, higher platelet count, higher CRP level, and lower albumin level in the initial phase of Kawasaki disease were strong risk factors of cardiac lesions. This information may help guide earlier clinical diagnosis of cardiac lesions using echocardiography. Early detection of cardiac lesions should lead to more appropriate treatment of Kawasaki disease. As such, early evaluation of initial clinical parameters is very important for managing patients with Kawasaki disease.

### Conclusions

Clinicians should consider sex, age, platelet count, albumin levels, and CRP levels when assessing risk of cardiac lesions in the initial phase of Kawasaki disease.

## ONLINE ONLY MATERIAL

Abstract in Japanese.
