# Supplementary material for: Cardiac Lesions and Initial Laboratory Data in Kawasaki Disease: a Nationwide Survey in Japan
Source: J Epidemiol. 2015 Mar 5;25(3):189–93. doi: 10.2188/jea.JE20140128 (PMC4340995; doi:10.2188/jea.JE20140128)
Supplement: Abstract in Japanese. [file je-25-189-s001.pdf]

川崎病初診時の心臓病変リスク因子：全国疫学調査

桑原政成[1,2]、屋代真弓[1]、小谷和彦[1]、坪井聡[1]、阿江竜介[1]、中村好一[1]、柳川洋[1]、川崎富作[3]
1 自治医科大学 公衆衛生学教室、2 虎の門病院 循環器センター内科、3 日本川崎病研究センター

背景：川崎病では冠動脈拡張、動脈瘤、心筋梗塞、弁膜病変などの心臓病変が時々認められる。これまでの川崎病における心臓病変の評価は、急性期から慢性期にかけて行われたものがほとんどである。本研究は各種血液検査データを含む川崎病初診時の状態と、心臓病変との関係性について明らかにした。
方法:本邦で2011年1月から2012年12月までに行われた､第22回川崎病全国調査の26691例の患者データを用いて横断研究を行った。本研究では、入院時に川崎病の症状出現から8日以内で､各種血液検査（白血球数､血小板数､血清アルブミン､C反応性タンパク（CRP））を行っている23155例（男児13353例、平均日齢：923±734日）を対象とした。
結果：心臓病変は984例（男児656例）に認められ、内訳は冠動脈拡張（764例）、冠動脈瘤（40例）、巨大冠動脈瘤（6例）、冠動脈狭窄（3例）、弁膜病変（204例）であった。冠動脈拡張の有意な関連因子は、加齢（100日増加ごとにオッズ比（OR）: 1.034）、男性（OR：1.731）、高血小板数（10000 /µL増加ごとにOR：1.006）、低アルブミン（1 g/ dL増加ごとにOR：0.659）、高CRP（1 mg/ dL増加ごとにOR：1.023）であった。冠状動脈瘤の有意な関連因子は、高血小板（10000 /µL増加ごとにOR：1.010）、低アルブミン（1 g/ dL増加ごとに OR：0.337）であった。巨大冠動脈瘤の有意な関連要因は認めなかった。弁膜病変の有意な関連因子は、日齢（100日増加ごとにOR：0.977）、高CRP（1 mg/ dL増加ごとにOR：1.045）であった。
結論：川崎病の初診時において、心臓病変を見逃さないために、男性、年齢、高血小板数、低アルブミン、高CRPに注意を払うことが必要である。

キーワード：川崎病、冠動脈疾患、冠動脈瘤、弁膜病変、疫学
